# Isolation, identification, and mechanism analysis of plant growth-promoting rhizobacteria in tobacco

**DOI:** 10.3389/fmicb.2024.1457624

**Published:** 2024-09-20

**Authors:** Chuandong Jiang, Fuyu Peng, Li Zhang, Yuqin Zhang, Jie Wang, Junmin Li, Binghui Cui, Changdai Cao, Chengqiang Wang, Yunlei Qin, Ran Wang, Zongpeng Zhao, Jiazhu Jiang, Mingfeng Yang, Mingming Sun, Long Yang, Qiang Zhang

**Affiliations:** ^1^College of Plant Protection, Shandong Agricultural University, Tai'an, China; ^2^Shandong China Tobacco Industry Co., Ltd., Jinan, China; ^3^Shandong Rizhao Tobacco Co., Ltd., Rizhao, China; ^4^College of Life Science, Shandong Agricultural University, Tai'an, China

**Keywords:** growth and development, microbiome and metagenomic analysis, plant growth-promoting rhizobacteria, tobacco, soil improvement

## Abstract

Plant growth, crop yield, and pest and disease control are enhanced by PGPR (Plant growth promoting rhizobacteria), which are beneficial microorganisms found in a close symbiosis with plant roots. Phytohormones are secreted, nutrient uptake is improved, and soil properties along with the microbiological environment are regulated by these microorganisms, making them a significant focus in agricultural research. In this study, the efficient PGPR strain T1 was isolated and screened from tobacco inter-root soil, and identified and confirmed by ITS sequencing technology. Tobacco growth indicators and soil property changes were observed and recorded through potting experiments. The activities of key enzymes (e.g., sucrase, catalase, urease) in soil were further determined. High-throughput sequencing technology was utilized to sequence the soil microbial community, and combined with macro-genomics analysis, the effects of T1 strain on soil microbial diversity and metabolic pathways were explored. Following the application of T1, significant improvements were observed in the height, leaf length, and width of tobacco plants. Furthermore, the physical and chemical properties of the soil were notably enhanced, including a 26.26% increase in phosphorus availability. Additionally, the activities of key soil enzymes such as sucrase, catalase, and urease were significantly increased, indicating improved soil health and fertility. Comprehensive joint microbiomics and macrogenomics analyses revealed a substantial rise in the populations of beneficial soil microorganisms and an enhancement in metabolic pathways, including amino acid metabolism, synthesis, and production of secondary metabolites. These increase in beneficial microorganisms and the enhancement of their metabolic functions are crucial for plant growth and soil fertility. This study provides valuable references for the development of innovative microbial fertilizers and offers programs for the sustainable development of modern agriculture.

## 1 Introduction

Tobacco (*Nicotiana tabacum* L.), belonging to the *Solanaceae* family and *Nicotiana* genus, is an annual herb. As a crucial pillar industry for China's economic development, tobacco has increased overall productivity. In recent years, large quantities of chemical fertilizers and pesticides have been applied to pursue economic benefits (Durham and Mizik, 2021). Tobacco growth and soil physical and chemical properties have been significantly deteriorated. Recently, the enhancement of tobacco productivity primarily relies on the extensive use of chemical fertilizers and pesticides. This dependence on chemical inputs has damaged the soil's physical and chemical properties and microenvironment. Researchers have found that plant growth-promoting rhizobacteria (PGPR) can effectively promote plant growth and improve soil quality, thereby reducing the dependence of tobacco agriculture on chemical fertilizers and pesticides (Khatoon et al., 2020). The rhizosphere refers to a minute region around plant roots distinct in their physical, chemical, and biological properties due to the influence of root activities. Microorganisms colonizing this area are known as rhizosphere microorganisms (Odelade and Babalola, 2019). PGPR are microorganisms that can extensively colonize or survive in the soil at the plant root surface (Martínez-Viveros et al., 2010). Unlike phylloplane microbes, which are located on the surface of plant leaves and are directly exposed to the external environment, endophytes reside within the internal tissues of the plant. Rhizosphere microorganisms are primarily found in the soil surrounding plant roots, with their interactions with the plant being mediated through substances secreted by the roots (Pii et al., 2015).

Researchers have found that PGPR directly or indirectly promotes plant growth, suppressing plant diseases and enhancing stress resistance (Basu et al., 2021). Additionally, soil physicochemical properties and microbial structure improvements were observed (Vocciante et al., 2022). Previous research indicates that PGPR synthesizes and releases phytohormones, including IAA, cytokinin, and gibberellins, which enhance root secretion and bolster plant resistance to abiotic stresses (Deng et al., 2023; Ulrich et al., 2021).

Through the metabolism and physiological activities of PGPR, chelated minerals in the soil are activated, making previously inaccessible nutrients available for reuse and creating conditions conducive to plant growth and development (Ha et al., 2014). For example, *Ochrobactrum haematophilum*, isolated from the inter-root of sweet potato, was found through metabolome and transcriptome analyses to provide phosphorus to plants under phosphorus-deficient conditions by secreting significant quantities of organic acids, thus participating in the phosphorus solubilization process (Ding et al., 2021).

The resistance of wheat to arid and semi-arid environments was enhanced through the release of potassium by *Bacillus megaterium*, which facilitated the production of extracellular polysaccharides and phytohormones (Rashid et al., 2022). Additionally, the growth and metabolism of pathogenic bacteria in the soil inter-root were inhibited by PGPR by producing antimicrobial substances, leading to intracellular lysis (Hammami et al., 2013). Concurrently, beneficial bacteria in PGPR compete for environmental resources such as nutrients, occupy favorable conditions for microbial growth and reproduction, and suppress the survival of harmful bacteria (Bhattacharyya and Jha, 2012).

Following the inter-root application of PGPG to plants, crop yields are increased, and modifications to the structure and function of the soil microbiota are induced (Ai et al., 2022). After applying PGPG, interactions occur among soil nutrients, enzymes, and microorganisms, enhancing the microecological conditions of the soil (Grover et al., 2021; Fiorentino et al., 2018). The conversion of soil nutrients is influenced, and as a result, microbial decomposition of soil organic matter (OM) leads to alterations in the content of effective N, P, and K in the soil and changes in soil enzymes. The effective soil nutrients and enzyme activities reflect the presence and activity of corresponding functional microorganisms, through which the microbial response mechanisms to environmental changes are evaluated (Nannipieri et al., 2002). Previous studies have shown that the community structure of indigenous soil microorganisms was altered to some extent by PGPR, and beneficial microorganisms capable of transforming soil nutrients were recruited and induced in the soil (Li et al., 2021). The co-application of PGPR and biochar enhanced soil sucrase activity, electrical conductivity (EC), and TK concentration. Significant reductions in catalase and superoxide dismutase activity and malondialdehyde levels were observed following the application of *Acinetobacter johnsonii*. Improvements in soil physiochemistry and the uptake of essential nutrients by maize were observed, and salt tolerance was conferred (Ren et al., 2021).

However, research on tobacco PGPR has been limited. Additionally, the effects of the interaction between tobacco and PGPR on the community and the composition of soil microorganisms are largely unknown. The microbiome includes microorganisms (bacteria, archaea, lower and higher eukaryotes, viruses), genomes, and surrounding environmental conditions (Marchesi and Ravel, 2015). The plant microbiome includes all microorganisms inhabiting the plant host, encompassing symbiotic, mutualistic symbioses, and harmful pathogens (Müller et al., 2016). From plant germination to maturation, the microbiome plays a critical role, interacting closely with the host (Berg et al., 2016). Potential diseases are warded off from plants by microorganisms that promote plant development (Pérez-García et al., 2011). Colonization sites for plant microorganisms have been classified into interleaf, inter-root, and endophytic microorganisms, with the diversity of inter-root microorganisms contributing to the stability of the soil ecosystem (Trivedi et al., 2020; Carrión et al., 2019). Therefore, studying the microbiome is crucial for understanding the mechanisms of action of PGPR on tobacco.

In addition, the market is dominated by bacterial bioagents, with fewer fungal bioagents available. Although significantly effective, bacteria are less stable and more susceptible to environmental influences (de Vries et al., 2018). Fungal bioagents are noted for their stronger environmental adaptability than bacterial bioagents, surviving under a broader range of conditions (Anwar and Shahnaz, 2022). Multiple biological control mechanisms, including the production of antibiotics, parasitic effects, and induction of plant resistance, are possessed by fungal bioagents, which are less likely to develop resistance (Admassie et al., 2019). Additionally, forming spores or other survival structures in the soil by fungal formulations provides a long-term effect. Consequently, the study of fungi-centered bioagents is particularly important.

This study successfully screened the highly effective PGPR *Trichoderma harzianum* from inter-root soil and applied it to tobacco soil. The effect of PGPR on tobacco growth was evaluated by measuring tobacco plants' growth indexes and root vigor. Further, soil enzyme activity and effective nutrients were measured by PGPR to evaluate the improvement of tobacco soil. The mechanism of PGPR on tobacco growth and development and soil improvement was deeply investigated by combining the joint analysis of the microbiome and macro genome. It provides a foundation and useful reference for developing new microbial fertilizers.

## 2 Materials and methods

### 2.1 Experimental materials

Variety for test: Use of baking tobacco variety K326.

Test soil: Continuous cropping soil, sourced from the tobacco station in Linqu, Weifang, with 4 years of continuous cropping, characterized by the following physicochemical properties: organic matter at 42.54 g/kg, alkaline hydrolyzable nitrogen at 53.97 mg/kg, available phosphorus at 45.71 mg/kg, available potassium at 178.5 mg/kg, and a pH of 6.40 (Table 1).

**Table 1:** Soil and strain tested.

**S. No**	**Parameters**	**Experimental result**
Soil samples	Organic matter	42.54 g/kg
	Alkaline hydrolyzable nitrogen	53.97 mg/kg
	Available phosphorus	45.71 mg/kg
	Available potassium	178.5 mg/kg
	pH	6.40
**Strain**		
	T1	*Trichoderma harzianum*

Experimental strains: The strains were screened from high-quality and healthy fields of soil and have probiotic properties such as phosphorus solubilization, potassium solubilization, IAA production, and cellulose reduction (Supplementary Table S2). The GenBank accession number(s) of nucleotide sequence(s) was PQ135054. The strains include: T1 was *Trichoderma harzianum*. Distilled water and commercial microbial preparation are designated as negative (CK1) and positive controls (CK2), respectively. The finished bacterial agent CK2 used the liquid microbial fertilizer “Infusion of Golden Liquid” provided by Fujian Sanmu Biotechnology Co., Ltd., the main component of which is the *jelly-like Bacillus* (Table 1).

### 2.2 Experimental design

The experiment was conducted in pots within the greenhouse of Dai Zong Campus, Shandong Agricultural University, featuring two control groups: negative control CK1 and positive control CK2. Bacterial suspensions were prepared by inoculating strains into LB (Luria-Bertani) broth. They were incubated in a shaker at 37°C and 180 rpm for 16 h to obtain the seed liquid, which was then adjusted to an OD600 of ~2.5. The diluted seed liquid was inoculated into 50 ml LB broth using a 2% inoculum rate for each 50 ml LB liquid medium. When tobacco seedlings in floating trays develop four cotyledons, select uniformly healthy plants for transplantation into pots. Each treatment receives the same dose of different microbial suspensions: dilute 1.5 ml of each suspension to 300 ml and irrigate the root zone of the seedlings. CK1 was added with an equal amount of water, CK2 was added with an equal amount of finished bacterial agent, and the experimental group was added with an equal amount of bacterial liquid. All the treatments were set up in six parallels.

### 2.3 Isolation and screening of functional bacteria in tobacco

Ten grams of healthy soil are taken and placed in a sterile Erlenmeyer flask, to which 90 mL of sterile water is added. The flask is placed in a constant temperature shaker and oscillates at 25°C and 200 rpm for 4 h. The resulting solution is diluted in a 10-fold serial dilution, and 100 μL of the diluted solution is spread evenly onto a PDA (Potato Dextrose Agar) plate. The inoculated PDA plates are incubated at 25°C in a constant temperature incubator for 48 h, and the growth of colonies is observed. Colonies with distinct morphologies are picked from the original plate and transferred to new PDA plates for further purification. The purified fungal colonies are suspended in sterile water containing 15% glycerol and stored at −80°C in an ultra-low temperature freezer.

### 2.4 Analysis of physiological indicators

According to “Tobacco Agronomic Traits Survey and Measurement Methods” (YC/T142-2010) (Dai et al., 2024), plant height, stem circumference, number of effective leaves, maximum leaf length, and leaf width of tobacco plants were measured at 5 d intervals after transplanting, and the maximum leaf area was calculated (leaf area = leaf length × leaf width × 0.6345). After 30 days, three representative plants per treatment were analyzed for dry matter accumulation by separating them into roots, stems, and leaves, measuring fresh weight before drying at 105°C for 30 min and then at 80°C until constant weight for dry weight recording. Root system metrics such as total length, surface area, diameter, and number of tips and branches were measured using a Microtek Phantom 9980XL scanner. Chlorophyll content was assessed every 5 days in the 3rd−4th cotyledons using a SPAD-502 meter.

### 2.5 Analysis of soil physical and chemical properties

After 30 d of growth, representative plants were selected for sampling, and three soil samples per treatment were mixed well to test the soil pH, alkaline hydrolyzable nitrogen (AN), available phosphorus (AP), available potassium (AK), and organic matter (OM) content. The pH meter was used to determine the soil pH, sodium bicarbonate extraction-molybdenum-antimony anti-spectrophotometric was used to determine AP (He et al., 2018), ammonium acetate-flame photometer was used to determine AK (Zhou et al., 2021), and alkaline dissolved nitrogen was used to determine AN (Nebbioso and Piccolo, 2011). The potassium dichromate volumetric method was used to determine OM (Zeyede, 2020).

### 2.6 Analysis of soil enzyme activity

Representative plants were selected for sampling after 30 d of tobacco plant growth, and three soil samples were taken from each treatment and mixed homogeneously using a kit (purchased from Beijing Prime Biological Co., Ltd.) to determine sucrase, urease, and catalase in the soil.

### 2.7 Extraction and functional analysis of soil microorganisms

#### 2.7.1 Sequencing of 16SrRNA and ITS gene amplicons of inter-root soil microorganisms

Genomic DNA was extracted from rhizosphere soil samples using the Omega E.Z.N.A™ Mag-Bind Soil DNA Kit. PCR amplification targeted the V3–V4 regions of the 16S rRNA gene using the universal primers 341F (CCTACGGGNGGCWGCAG) and 805R (GACTACHVGGGTATCTAATCC), as well as the ITS1-ITS2 regions using the universal primers ITS1F (CTTGGTCATTTAGAGGAAGTAA) and ITS2R (GCTGCGTTCTTCATCGATGC) (Coenye et al., 1999). The library size was verified by 2% agarose gel electrophoresis, and the library concentration was determined using a Qubit 4.0 fluorescence quantifier to ensure consistent long cluster results and high-quality sequencing data. Sequencing was conducted on the Illumina MiSeq platform.

#### 2.7.2 Analysis of amplicon sequence processing and bioinformatics

Sequencing data were processed on the BioSignal Cloud Platform (https://ngs.sangon.com/). The process involved removing primer sequences, filtering out low-quality sequences, and splicing short pairs of reads from double-end sequencing into single sequences using Flash. These spliced reads were further refined to obtain clean reads. Sequences were restricted to ≥97% similarity, and non-repetitive sequences were clustered into operational taxonomic units (OTUs) using UPARSE (version 7.0.1090). To assess α-diversity, indices such as Chao 1, Ace, Simpson, Shannon, Shannoneven, and Sobs were calculated using Mothur software (Nossa et al., 2010). To visualize changes in microbial composition, principal component analysis (PCA) was conducted on treated samples using QIIME and R software (v. 3.5.3) (Abdi and Williams, 2010). Additionally, redundancy analysis (RDA) was conducted to identify environmental factors, with variance inflation factor (VIF) analysis used for screening (Chen et al., 2019).

#### 2.7.3 Soil macro-genome sequencing

Genomic DNA was extracted from the inter-root soil samples using the OMEGA kit E.Z.N.A™ Mag-Bind Soil DNA Kit, and the DNA was quantitatively analyzed using a Qubit 4.0 fluorescence quantifier Ltd. for sequencing on the Illumina MiSeq sequencing platform to obtain a large amount of raw data. Megahit software was used to perform multi-sample hybrid splicing of clean reads; SPAdes software was used to perform hybrid splicing of unmatched reads, and fragments ≤ 500 pb were filtered for downstream analyses, such as statistics and subsequent gene prediction. DIAMOND was used to compare the gene set protein sequences with the KEGG database, to obtain the corresponding KO numbers of the sequences, and to count the abundance of each functional level of KEGG in each sample. The sequencing results were compared with the KEGG (http://www.kegg.jp/kegg/) database using DIAMOND software at default values to predict microbial metabolic functions.

### 2.8 Data analysis

The experimental data were counted and graphed using Microsoft Excel 2021 and Origin 2023 and analyzed for the significance of differences using Duncan's test with SPSS Statistics 25.0 software.

## 3 Results

### 3.1 Genetic characterization of the PGPR

Sequence comparison was conducted using the Blast tool in the NCBI database, and a phylogenetic tree was constructed using Mega11 software (Supplementary Figure S1). The analysis revealed that strain T1 resides on the same minimal branch as *Trichoderma harzianum*, indicating the closest evolutionary distance. Based on ITS genome sequence analysis and physiological and biochemical assessments, the strains were preliminarily identified as *Trichoderma harzianum*.

### 3.2 Effect of PGPR on agronomic traits of tobacco

**Figure 1:**
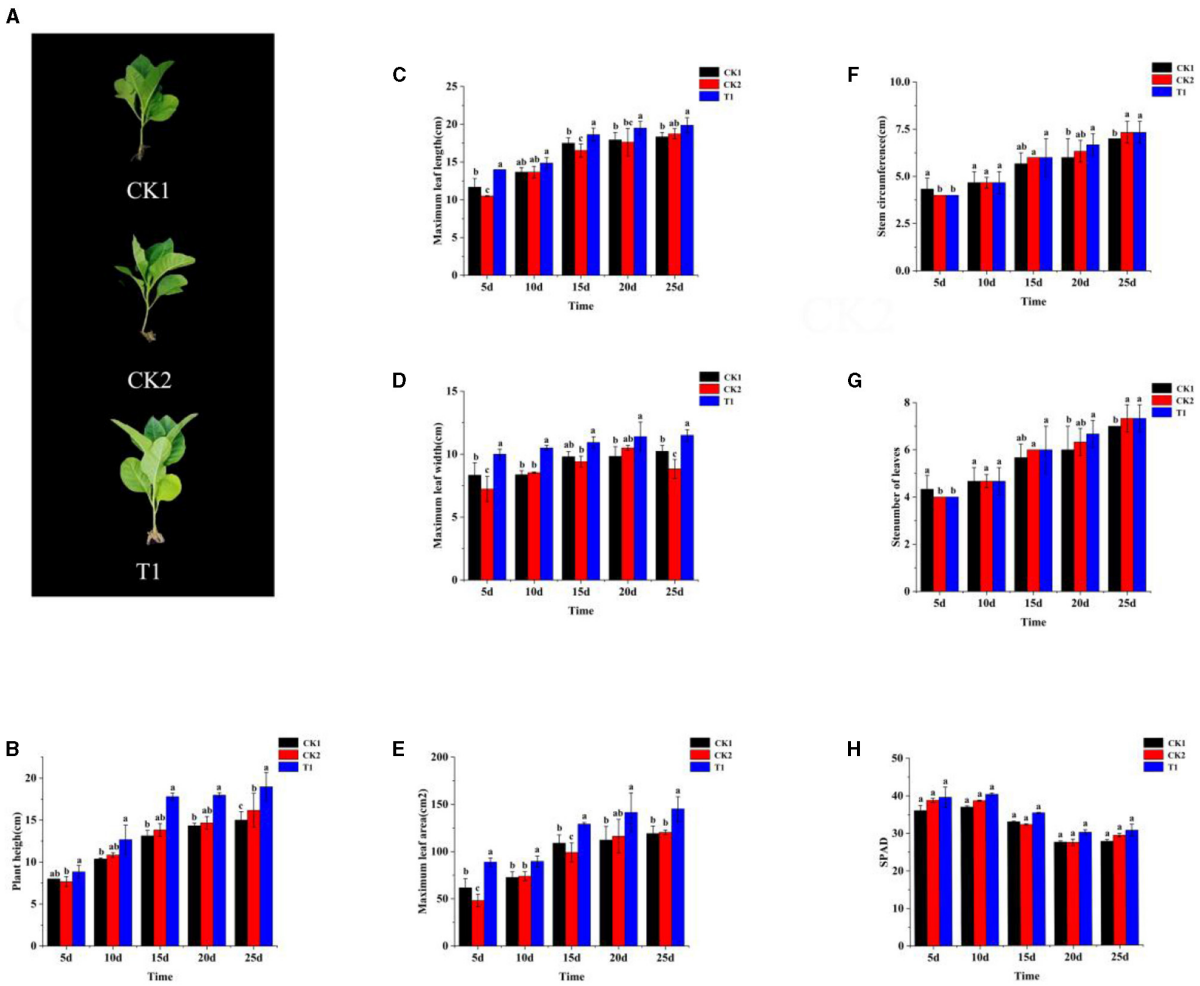
Agronomic traits of tobacco **(A)** phenotype, **(B)** plant height, **(C)** maximum leaf length, **(D)** maximum leaf width, **(E)** maximum leaf area, **(F)** stem circumference, **(G)** Stenumber of leaves, and **(H)** SPAD. Small letters in the bar chart mean significant difference among different treatments (*P* < 0.05).

To verify the growth-promoting effect of the PGPR on tobacco, the screened fungi were tested in pots. All treatments with microbial inoculum exhibited a pro-vigorant effect under continuous soil conditions compared to the CK. At 30 days after transplanting, tobacco phenotypes were assessed under four different application conditions, and all treatments with mycorrhizal fungi showed better growth (i.e., bigger and more leaves, taller plants) than CK1 (Figure 1A). Regarding leaf length, all microbial inoculum treatments promoted tobacco leaf length, with the T1 treatment significantly outperforming the other treatments throughout the growing period (Figure 1B). Moreover, 10 days after transplanting, plant height, leaf length, width, and maximum leaf area were significantly higher in the T1 treatment compared to the CK (Figures 1B–E). However, there were no significant differences between T1 and CK in terms of stem circumference and the number of effective leaves. While there were observable differences between treatments in stem circumference and the number of leaves, these differences did not reach a significant level (Figures 1F, G).

The effects of different microbial inoculums on SPAD values of baked tobacco leaves showed significant differences (Figure 1H). At 5 days after transplanting, SPAD values for all treatments with microbial inoculums were significantly higher than those for CK1, with the T1 treatment showing a particularly significant difference compared to CK1. At 10 days after transplanting, the SPAD values for CK2 and T1 treatments showed a significant advantage, increasing by 5.62 and 7.96%, respectively, compared to CK1. Although the chlorophyll content in each treatment decreased 15 days after transplanting, the SPAD values for the T1 treatments remained significantly higher than those of CK1. By 20 days after transplanting, the SPAD value of T1 was significantly higher than all other treatments, with an increase of 6.15% compared to CK1, and there was no significant difference between CK1 and CK2. At 25 days after transplanting, the difference between T1 and CK1 remained significant.

### 3.3 Effect of PGPG on the root system of tobacco

**Figure 2:**
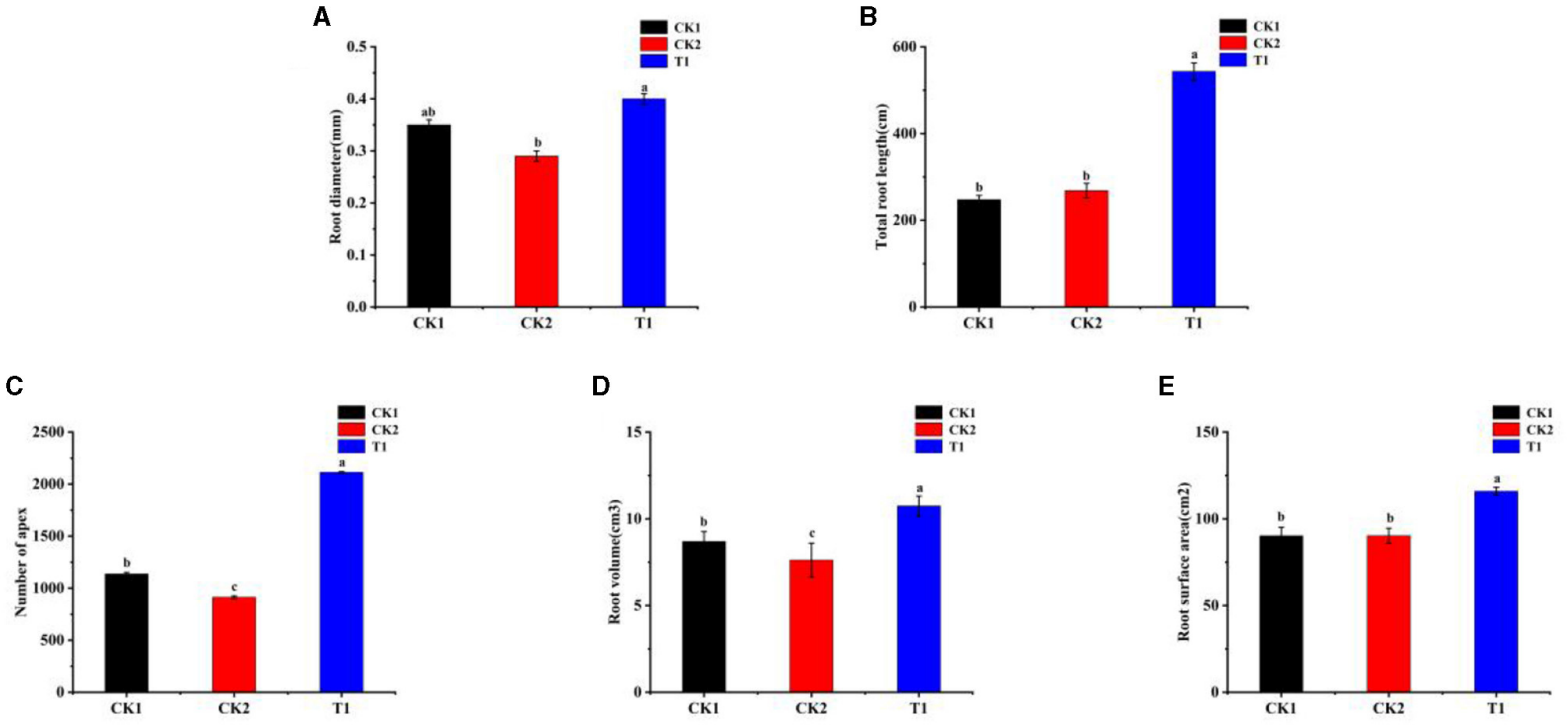
Root system **(A)** root diameter, **(B)** total root length, **(C)** number of apex, **(D)** root volume, and **(E)** root surface area. Small letters in the bar chart mean significant difference among different treatments (*P* < 0.05).

During the seedling stage, the role of roots is considered far more significant than that of leaves. Initially, the survival and early growth of the plant rely heavily on a robust root system, especially during the seedling stage, where roots form the basis for the absorption of water and nutrients (Jackson et al., 2007). A healthy root system effectively absorbs water and nutrients from the soil, providing the foundation for rapid plant growth (Fageria and Moreira, 2011). In contrast, although leaves are the primary sites for photosynthesis and the production of economic value in tobacco, their function is not fully utilized during the seedling stage. Therefore, the root parameters of tobacco were measured. There were significant differences in the effects of different treatments of PGPR on the growth and development status of tobacco roots. The difference between CK2 and CK1 was not significant. The results showed that the root diameter of T1 treatment was higher than that of CK1, which was 14.2% higher than that of CK1 (Figure 2A).

Regarding the total root length of the root system, the T1 treatments showed a significant advantage, which increased by 83.31% compared with CK1 (Figure 2B). For the number of root tips in the root system, T1 was increased by 67.20% compared with CK1 (Figure 2C). In addition, the T1 treatment had a significant advantage of 29.69% compared with CK1 (Figure 2D). Meanwhile, CK2 treatments were smaller than CK1 and reduced by 12.31%. Compared with CK1, root surface area was significantly increased in all treatments, and T1 treatment was significantly higher than other treatments and increased by 18.65% compared with CK1 (Figure 2E).

### 3.4 Effect of PGPR on tobacco biomass

**Figure 3:**
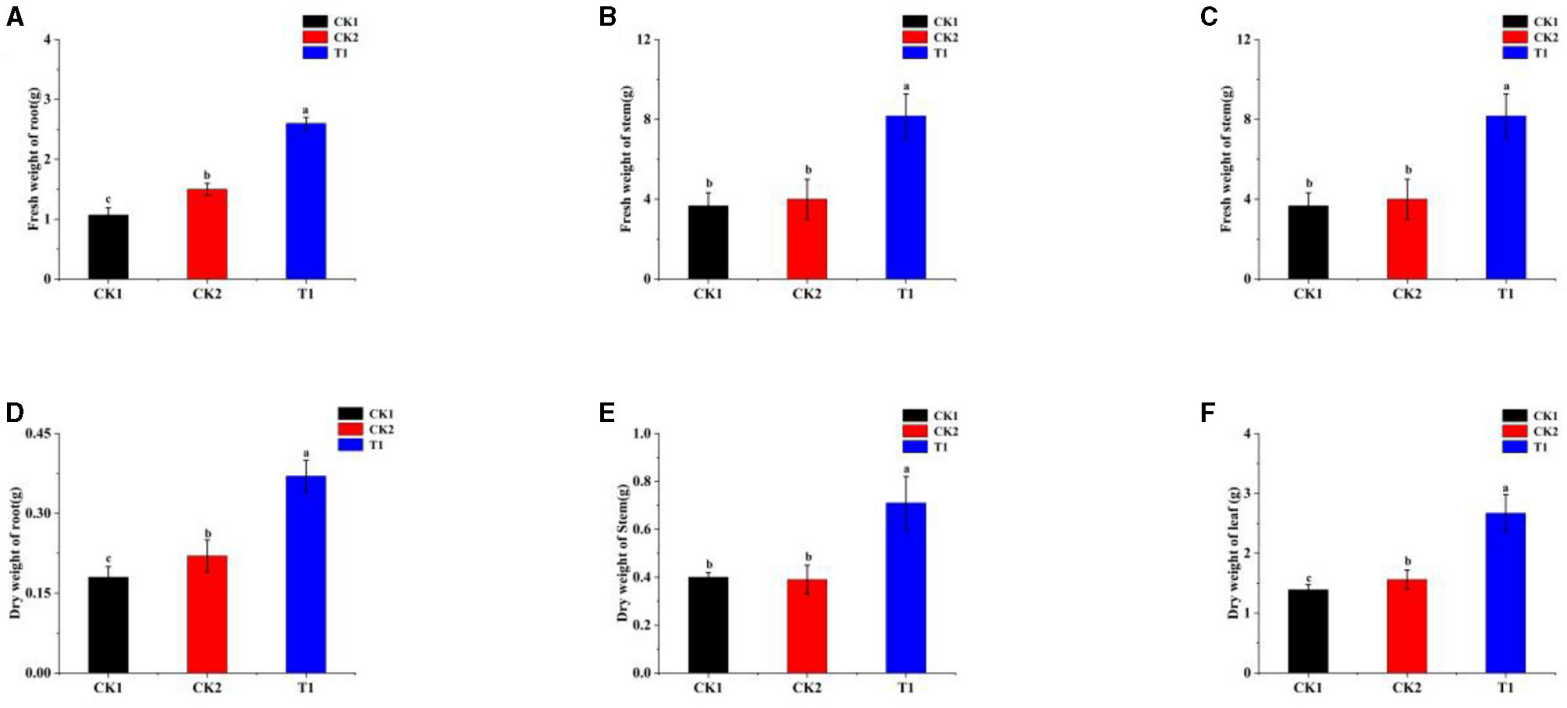
Substance accumulation **(A)** fresh weight of root, **(B)** fresh weight of stem, **(C)** fresh weight of leaf, **(D)** dry weight of root, **(E)** dry weight of stem, and **(F)** dry weight of leaf. Small letters in the bar chart mean significant difference among different treatments (*P* < 0.05).

The effects of different treatments on the accumulation of roasted tobacco substances showed significant differences. The T1 treatment was significantly superior to the other treatments for root fresh weight, with a 142.99% increase over CK1 (Figure 3A). T1 treatment showed a significant advantage for stem fresh weight, and all stem fresh weights of microbial-agent applied treatments were higher than that of control CK1 (Figure 3B). Regarding leaf fresh weight, the T1 treatment was particularly outstanding, with a 77.04% increase compared with CK1, which was significantly better than the other treatments (Figure 3C). In addition, the root dry weight of the T1 treatment also demonstrated a significant advantage with a 105.56% increase over CK1, significantly different from other treatments (Figure 3D). For stem dry weight, all treatments except CK2 treatment were higher than CK1, especially T1, which was 77.50% higher than CK1 (Figure 3E). T1 treatment also showed a significant difference in leaf dry weight compared with other treatments, increasing 92.09% compared with CK1, and all treatments had higher leaf dry weight than CK1 (Figure 3F).

### 3.5 Effect of PGPR on soil physicochemical properties and enzyme activities

**Figure 4:**
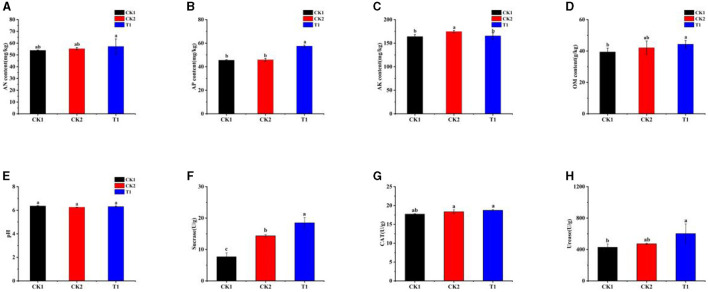
Soil physicochemical properties and enzyme activities **(A)** AN, **(B)** AP, **(C)** AK, **(D)** OM, **(E)** pH, **(F)** sucrase, **(G)** catalase, and **(H)** urease. Small letters in the bar chart mean significant difference among different treatments (*P* < 0.05).

Carbon, nitrogen, phosphorus levels, and soil enzyme activities are key indicators of soil fertility. To assess these factors, we investigated changes in soil properties and the activities of essential soil enzymes following the application of microbial inoculums. The measurements included dissolved alkaline nitrogen (AN), organic matter (OM), quick-acting phosphorus (AP), quick-acting potassium (AK), and soil pH. The results showed no significant difference in AN content between treatments (Figure 4A). However, the AP content in the T1 treatment was significantly higher than in other treatments, showing a 26.26% increase compared to the CK1 treatment (Figure 4B). While there was no significant difference in AK content across treatments, all treatments exhibited higher AK levels than CK1 (Figure 4C). The T1 treatment also significantly increased OM content in the soil by 14.48% compared to CK1, with a significant difference (Figure 4D). Regarding soil pH, all treatments led to a decrease in pH levels (Figure 4E).

Second, the activities of SU, CAT, and UR in the test soils were measured, revealing significant differences in enzyme activities among the treatments. All treatments involving the applied strain showed higher than SU activity than CK1 (Figure 4F). Specifically, the SU activity in the T1 treatment was significantly higher than in other treatments, showing a remarkable increase of 140.13% compared to CK1. Although the effect of different treatments on CAT activity in the soil was not significantly different, the highest CAT activity was recorded in the T1 treatment, with an increase of 5.59% compared to CK1 (Figure 4G). UR activity in the soil was also significantly affected by the different inter-root biotrophic bacteria. Both CK2 and T1 showed higher UR activity content than CK1, with T1 significantly boosting UR activity by 40.25% compared to CK1 (Figure 4H).

### 3.6 Effect of PGPR on microbial diversity

#### 3.6.1 Analysis of high-throughput sequencing results and out clustering

To verify the sequencing quality and sequencing depth, the reliability of the sequencing data volume was assessed by the dilution curve. As the number of sequencings increased, the dilution curve gradually smoothed out, and the value became higher and higher and tended to be 1 (Supplementary Figures S2A, B). This indicated that most of the species in the soil samples were detected, that more data volume would only generate a small number of new OTUs, and that the sequencing data was sufficient. The results were representative of the real situation of the samples. They could be used for the subsequent analysis of the diversity of the soil community and the composition of the species.

The distribution of species community-specific and shared OTUs is shown by the Venn diagram. The inter-root bacterial community had 3,634 OUTs in the two treatments, whereas 599 specific OUTs were specific to T1, and 796 were specific to the CK. The fungal community had 2,082 OUTs in the two treatments, while 481 and 463 specific OUTs were specific to CK and T1, respectively (Supplementary Figures S2C, D).

To explore the differences and distances in the composition of bacterial and fungal communities in different treatments, PCA analysis of bacterial and fungal communities in different treatments was performed based on OTU level. The PC1 and PC2 axes of the bacterial community were segregated at 0.087 and 0.76%, respectively. The T1 treatment was dispersed individually compared to CK, indicating differences in species composition among the treatments. The fungal community had 0.055 and 0.901% PC1 and PC2 axes segregation rates, respectively, and were relatively dispersed among treatments with differences in species composition structure (Supplementary Figures S2E, F).

#### 3.6.2 Effect of alpha and beta diversity of soil microorganisms

**Figure 5:**
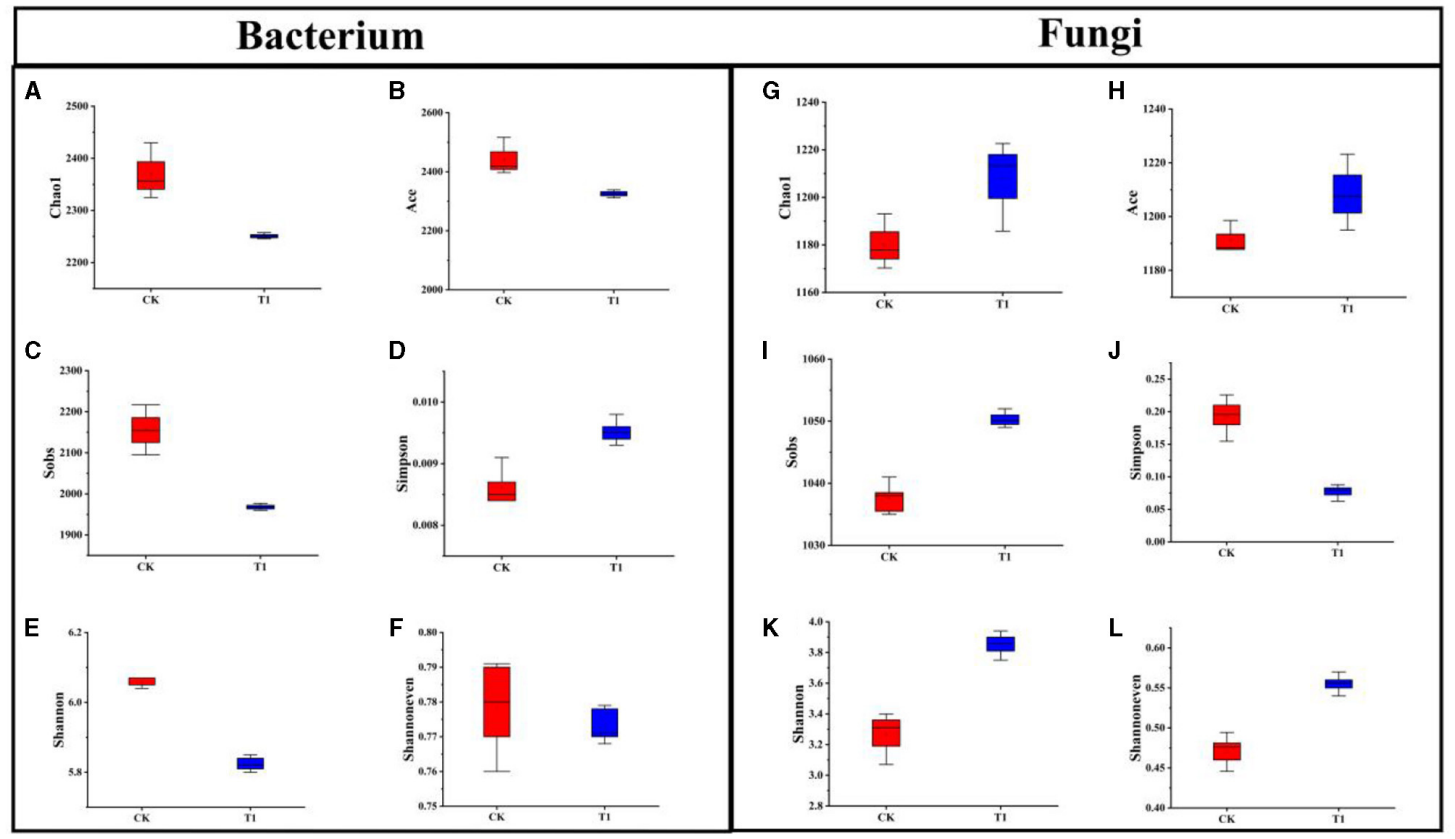
Alpha-diversity index of rhizosphere bacteria **(A)** Chao1, **(B)** Ace, **(C)** Sobs, **(D)** Simpson, **(E)** Shannon, **(F)** Shannoneven. Alpha-diversity index of rhizosphere fungi **(G)** Chao1, **(H)** Ace, **(I)** Sobs, **(J)** Simpson, **(K)** Shannon, **(L)** Shannoneven.

The effect of different treatments on the α-diversity of inter-root bacteria, Chao1, and Ace indices decreased in the T1 treatment compared to CK, indicating that the T1 treatment decreased the relative abundance of inter-root bacteria in the microbial community (Figures 5A, B). Sobs index was higher in CK than in T1 treatments, suggesting that the actual number of OUTs was the highest in the CK treatment (Figure 5C). The increase in Simpson's index and decrease in Shannon's index in the T1 treatment compared to CK indicated that the community diversity of inter-root bacteria was reduced in the T1 treatment (Figures 5D, E). The Shannoneven index was higher in CK than in the T1 treatment, indicating that the uniformity of the inter-root bacterial community was higher in the CK treatment than in the T1 treatment (Figure 5F).

The effects of different treatments on the α-diversity of inter-root fungi were significant. The Chao1 and Ace indices of T1 increased compared to CK, indicating that the T1 treatment increased the number of OUTs of inter-root fungi in the community and improved the relative abundance of fungal flora in the microorganisms (Figures 5G, H). The Sobs index of T1 was greater than that of the CK control treatment, suggesting that the number of OUTs observed in the CK treatment was less than that of T1 (Figure 5I). Whereas, the Simpson's index of T1 declined compared to that of CK, the Shannon index increased, indicating that the T1 treatment increased the community's diversity (Figures 5J, K). The Shannoneven index of the CK treatment was smaller than that of the T1 treatment, indicating that the T1 treatment had a high uniformity in the distribution of the inter-root fungal flora (Figure 5L).

#### 3.6.3 Effect of PGPR on microbial composition and structure

**Figure 6:**
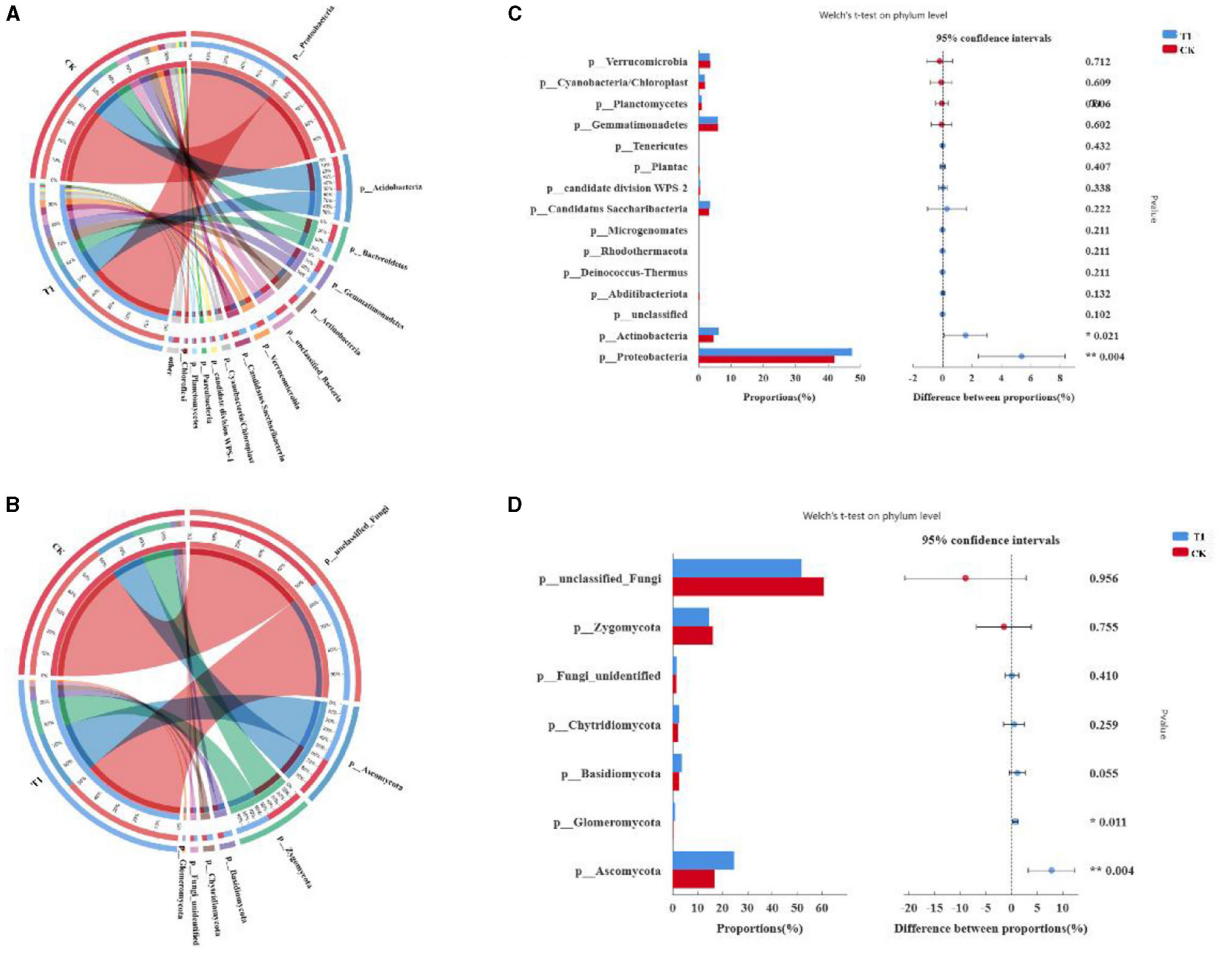
Analysis of microbial phylum level **(A)** composition and relative abundance of bacteria, **(B)** fungal, **(C)** comparative analysis of phylum level bacterial community, and **(D)** fungal.

To clarify the effect of different treatments on the species composition of microbial communities, changes in dominant species of bacteria and fungi were comparatively analyzed at the phylum level (Figures 6A, B), and the sum of species with relative abundance < 0.01 was defined as Others. The dominant species at the phylum level of bacteria were *Proteobacteria, Acidobacteria, Sphingomonas, Gemmatimonadetes, unclassified_Bacteria, Candidatus, Gp3, Saccharibacteria, Chitinophagaceae, Subdivision3, and Rhizobiales*. The most dominant phyla are *Rhizobiales, Gp1, Betaproteobacteria, Streptophyta, Bradyrhizobium, Alphaproteobacteria, and Acidobacterium*. *Proteobacteria, Acidobacteria*, and *unclassified_Bacteria* are the most dominant phyla. The average relative abundance was 7.55, 5.48, and 5.44%, respectively. The highest relative abundance of dominant species at the level of fungal phyla was *unclassified_Fungi, Mortierella, Ascomycota, Sordariomycetes, Alternaria, Trichoderma, Fungi_unidentified, Ascomycota, Ascomycota*. Among them, *unclassified_Fungi, Mortierella*, are the most dominant phyla. The average relative abundance was 63.2, 15.8, and 5.44%, respectively.

Comparative analyses of bacteria and fungi were performed to further explore species composition differences between treatments at the phylum level, comparing bacterial and fungal species with *P*-values in the top 15 and fungal species with *P*-values in the top 7. Compared with CK (Figures 6C, D), the relative abundance of *Proteobacteria, Candidatus Saccharibacteria*, and *Actinobacteria* was increased, and *Verrucomicrobia, Gemmatimonadetes*, and *Cyanobacteria/Chloroplast* were decreased by T1 in the bacterial community. Species composition of the fungal community differed at the phylum level; the relative divisions of Chytridiomycota*, Ascomycota, and Basidiomycota* were increased, and the relative divisions of *unclassified_Fungi, Zygomycota*, were decreased by T1 compared to CK.

**Figure 7:**
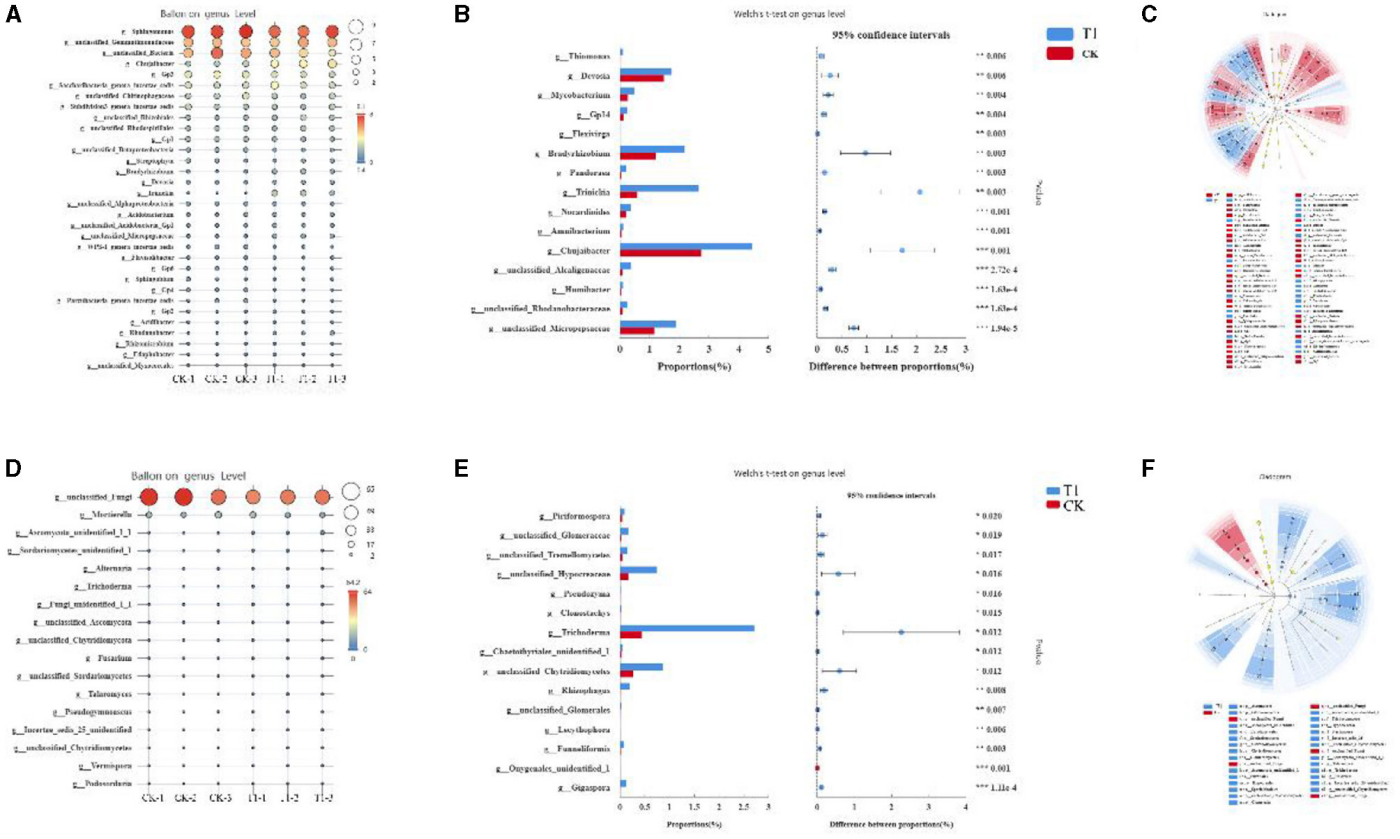
Analysis of microbial genus level **(A)** species composition analysis of dominant bacteria, **(B)** fungal, **(C)** comparative analysis of genus level bacterial community, **(D)** fungal, **(E)** LEfSe analysis results of bacterial; and **(F)** fungal.

The species composition of bacteria and fungi in different treatments at the genus level was analyzed. The dominant genera in the bacterial community were mainly *Sphingomonas, Gemmatimonadaceae, unclassified_Bacteria, Chujaibacter, Gp3, and Saccharibacteria* (Figure 7A). The dominant genera in the fungal community at the genus level were mainly *unclassified_Fungi, Mortierella, Sordariomycetes_, Ascomycota, Alternaria, and Trichoderma* (Figure 7B). Analysis of the differences in species composition of bacterial and fungal communities between treatments at the genus level showed that at the bacterial genus level, the relative abundance of the genera *Devosia, Mycobacterium, GPl4, Bradyrhizobium, Alcaligenaceae, Trinickia, Chujaibacter, Rhodanobacteraceae*, and *Micropepsaceae* was significantly increased by T1 (Figure 7C). For the fungal genus level, T1 increased the relative abundance of *Hypocreaceae, Trichoderma, and Chytridiomycetes* (Figure 7D).

To investigate further the differences in the composition of bacterial and fungal communities in the inter-root soil of tobacco under different treatments, linear discriminant analysis and influence factor (LEfSe) were used to discover the species that best explained the differences between groups under different treatments. Data from inter-root soil samples of tobacco plants were analyzed and counted at five levels from phylum to genus. A total of 55 different levels of differential taxa were identified in the bacterial community. A total of 32 differential species were enriched in the CK treatment group and 21 in the T1 treatment group (Figure 7E). In the bacterial community, for the phylum level, the five enriched taxa in CK are *p_Acidobacteria, p_Bacteroidetes, p_Chloroflexi, p_Parcubacteria, and p_unclassified_Bacteria*. The two enriched taxa in T1 are *p_Actinobacteria and p_Proteobacteria*. For the class level, the seven enriched taxa in CK are *c_Acidobacteria_Gp3, c_Acidobacteria_Gp4, c_Acidobacteria_Gp6, c_Chitinophagia, c_norank_Parcubacteria, c_Deltaproteobacteria*, and *c_Deltaproteobacteria*. The seven enriched taxa in T1 are *c_Actinobacteria, c_Betaproteobacteria, and c_Gammaproteobacteria*. For the order level, the eleven enriched taxa in CK are *o_Acidobacteria_Gp3, o_Acidobacteria_Gp4, o_Acidobacteria_Gp6, o_Chitinophagales, o_Parcubacteria, o_Sphingomonadales, o_Alphaproteobacteria, o_Nitrosomonadales, o_Betaproteobacteria, o_Myxococcales*, and *o_unclassified_Bacteria*. The six enriched taxa in T1 are *o_Micrococcales, o_Micropepsales, o_Rhizobiales, o_Burkholderiales, o_Gammaproteobacteria*, and *o_Xanthomonadales*. For the family level, the eight enriched taxa in CK are *f_Acidobacteria_Gp3, f_Acidobacteria_Gp4, f_Acidobacteria_Gp6, f_Chitinophagaceae, f_Sphingomonadaceae, f_Alphaproteobacteria, f_Betaproteobacteria*, and *f_unclassified_Bacteria*. The seven enriched taxa in T1 are *f_Micropepsaceae, f_Bradyrhizobiaceae, f_Devosiaceae, f_Burkholderiaceae, f_Gammaproteobacteria, f_Rhodanobacteraceae*, and *f_Xanthomonadaceae*. For the genus level, the six enriched taxa in CK are *g_Gp1, g_Gp3, g_Gp4, g_Flavisolibacter, g_Parcubacteria*, and *g_unclassified_Bacteria*. The six enriched taxa in T1 are *g_Micropepsaceae, g_Bradyrhizobium, g_Devosia, g_Trinickia, g_Acidibacter, g_Rhodanobacter*, and *g_Chujaibacter*.

A total of 31 taxa with different levels of differentiation were identified in the fungal community. There were five and 26 differential species in CK and T1, respectively. The enriched taxa of CK phylum, order, family, and genus are *unclassified_Fungi* (Figure 7F). For the phylum level, there were two enriched taxa in T1, namely *Ascomycota Glomeromycetes*. For the order level, there are six enriched taxa in T1, which are *p_Ascomycota, p_Eurotiales, p_Sordariomycetes, p_Microbotryomycetes, p_Chytridiomycetes*, and *p_Glomeromycetes*. For the order level, there are four enriched taxa in T1, which are *o_Ascomycota, o_Eurotiales, o_Hypocreaceae, o_Sporidiobolales, o_Chytridiomycetes, o_Glomerales*. For the family level, the six enriched taxa in T1 are *f_Ascomycota, f_Trichocomaceae, f_Hypocreaceae, f_Nectriaceae, f_Incertae_sedis_25*, and *f_Chytridiomycetes*. For the genus level, the four enriched taxa in T1 are *g_Ascomycota, g_Talaromyces, g_Trichoderma, g_Fusarium, g_Incertae_sedis_25*, and *g_Chytridiomycetes*.

#### 3.6.4 Correlation analysis of soil physicochemical and microbial diversity

**Figure 8:**
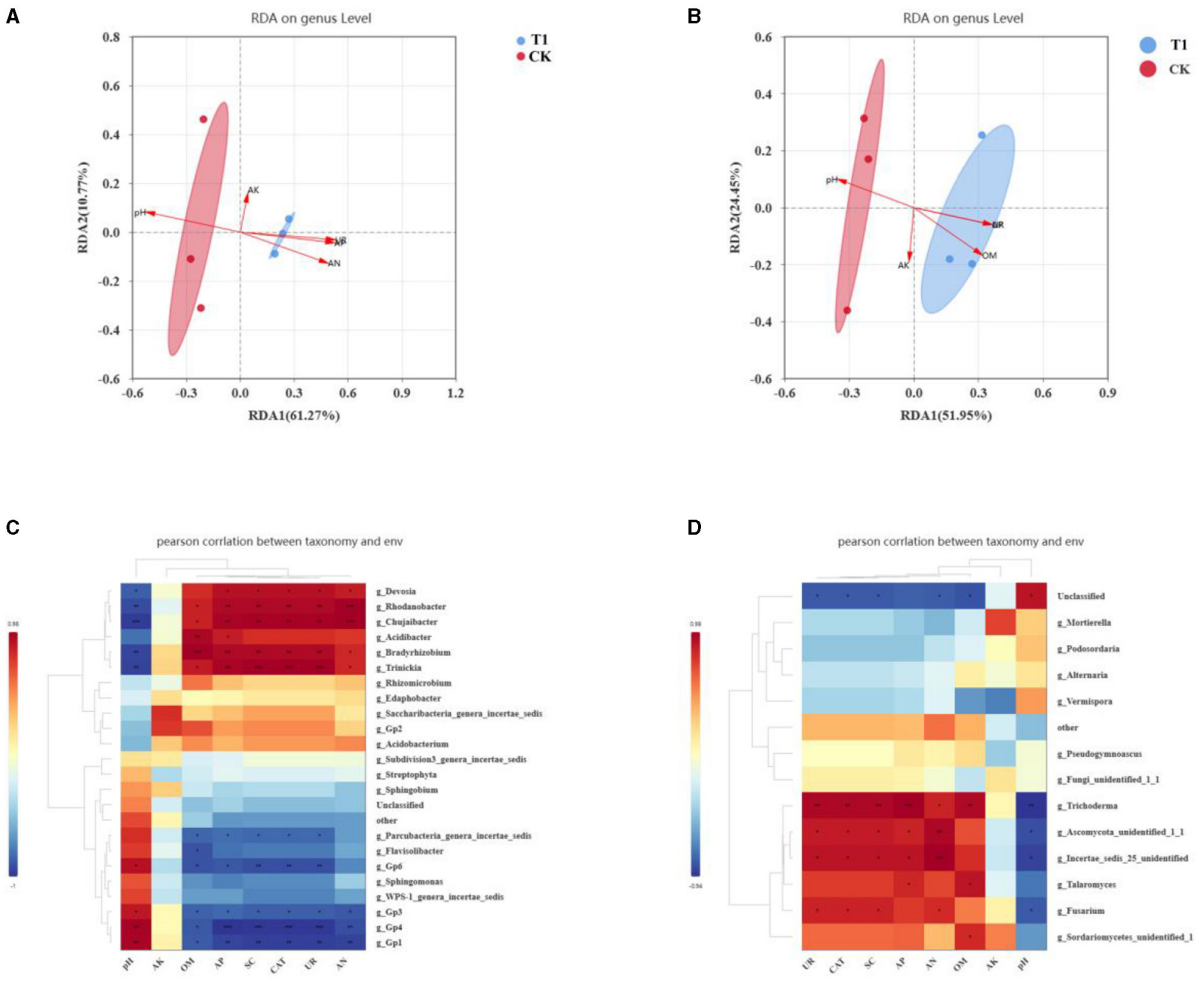
Correlation analysis **(A)** RDA analysis of nutrient and enzyme activities in rhizosphere bacterial, **(B)** fungal, **(C)** correlation of soil nutrient and enzyme activity with dominant genera of bacteria, and **(D)** fungal.

The addition of different inter-root biotrophic bacteria affects nutrient transformations in the soil and alters soil enzyme activities, contributing to changes in the microbial community in the soil. Therefore, the relationship between soil bacterial and fungal communities and environmental factors was investigated. Redundancy (RDA) analysis was used to correlate the physicochemical properties such as pH, AN, OM, AP, AK, and soil enzyme activities such as SC, CAT, and UR with the microbial communities in the soil (Figures 8A, B). The results showed that soil physicochemical property indexes, AN AP, and OM in soil were most affected by bacterial community, followed by OM. The effect of T1 treatment on AN, AP, and OM in soil was significantly positively correlated, and the correlation with AP was more significant.

Meanwhile, pH was significantly and negatively correlated with the T1 treatment. T1 treatment was significantly and positively correlated with SC and CAT in soil and not significantly with UR. By analyzing the effect of the fungal community on soil nutrients and enzyme activities, we found that AN, OM, and pH soil nutrients had the greatest effect on the soil, and the least correlation was with OM. The T1 treatment became significantly positively correlated with AN and AP and significantly negatively correlated with pH. Regarding soil enzyme activities, UR was most affected by fungal communities. T1 treatment was positively correlated with SC, CAT, and UR in soil, and UR was significantly correlated.

To further investigate the relationship between microbial community composition and soil nutrients and enzyme activities, a correlation heat map analysis was conducted at the genus level for dominant bacterial and fungal communities in relation to soil environmental factors (Figures 8C, D). In the bacterial community, genera such as *Devosia, Rhodanobacter, Chujaibacter, Bradyrhizobium, Trinickia, Gp6, Gp3, Gp4, Gp1*, and *Parcubacteria* played significant roles. These genera were positively correlated with soil physicochemical properties such as alkaline nitrogen (AN), available phosphorus (AP), organic matter (OM), sucrase (SC), and catalase (CAT) activities while being negatively correlated with soil pH. Conversely, Gp6, Gp3, Gp4, Gp1, and *Parcubacteria* showed significant negative correlations with AN, AP, OM, SC, and CAT and positive correlations with soil pH. There was no significant correlation between available potassium (AK) and the dominant bacterial genera. In the fungal community, genera such as *Trichoderma, Ascomycota*, and *Fusarium* were significantly positively correlated with AN, AP, AK, SC, and CAT activities. Specifically, *Trichoderma* showed a positive correlation with OM in the soil, while *Ascomycota* and *Fusarium* were negatively correlated with AN, AP, SC, UR, and CAT. Soil pH was negatively correlated with *Trichoderma*. Similar to the bacterial community, no significant correlation was found between AK and the dominant fungal genera.

#### 3.6.5 Effects of functional properties in soil microbial communities

**Figure 9:**
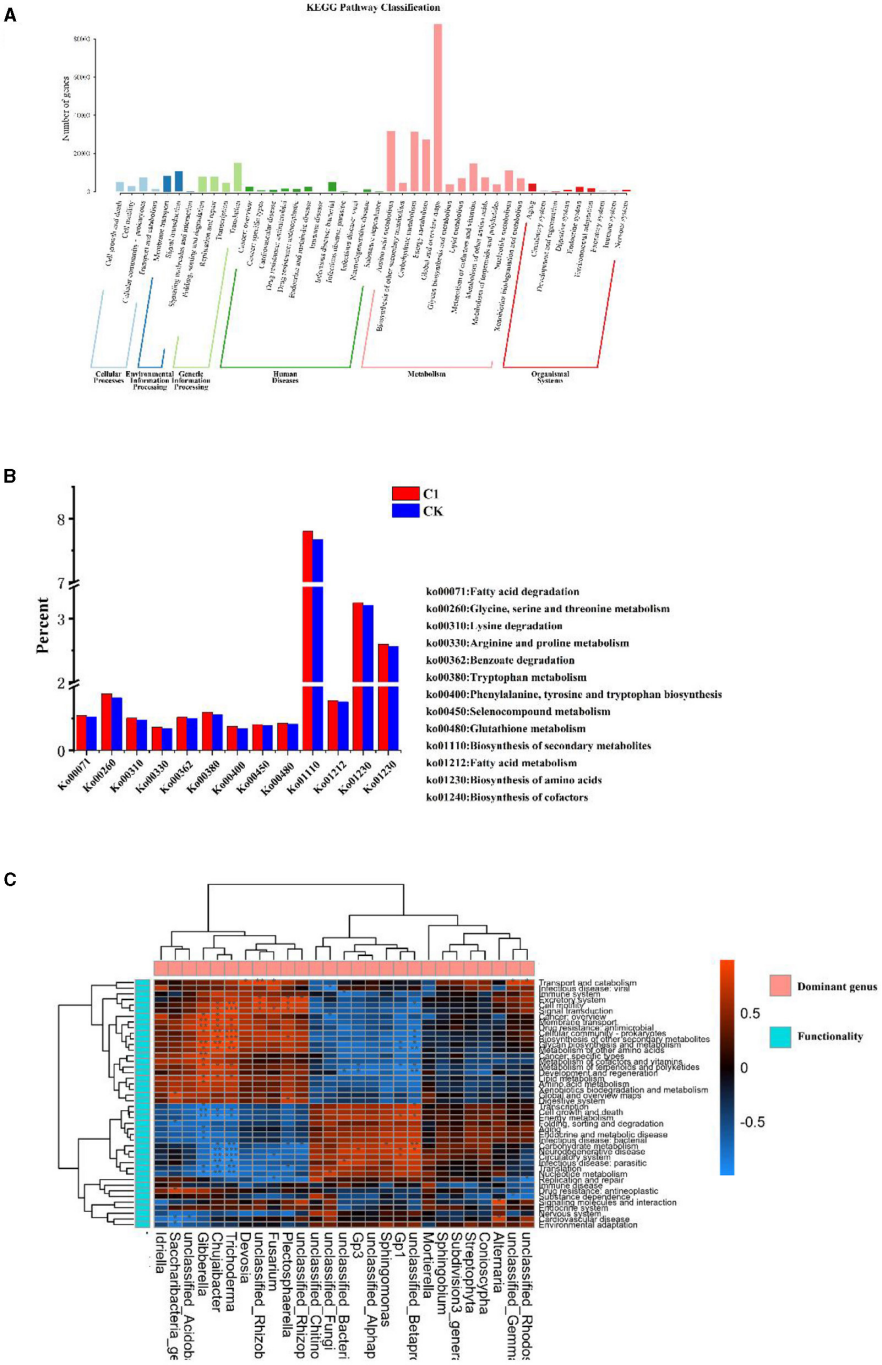
Analysis of metagenome **(A)** histogram of the number of genes at KEGG Pathway1 and Pathway2 levels, **(B)** influence of rhizosphere growth-promoting bacteria on the metabolic level of soil microorganisms KEGG Pathway3, and **(C)** heat map analysis of the correlation between dominant genera and KEGG function in soil.

Soil microbial metabolic functions, analyzed at both Pathway Level 1 and Level 2, reveal that metabolism is the most dominant activity among the six KEGG metabolic functions, highlighting its critical role in the life processes of microorganisms (Figure 9A). Within the KEGG Pathway database, the “global and overview maps” category, which encompasses a special set of metabolic pathway maps, contained the largest number of genes related to metabolic functions. The most prominent metabolic pathways identified were amino acid, carbohydrate, energy, cofactors, and microbial and nucleotide metabolism. In the domain of cellular processes, the “cell communities – prokaryotes” pathway had the highest gene count, while “transmembrane transport” and “signaling” pathways topped the environmental information processing category. Within gene information processing, the pathway with the most genes was related to signaling. Furthermore, the highest number of genes associated with bacterial infectious diseases were found in disease pathways. In the organismal systems pathways, “aging,” “endoanalytic systems,” and “environmental adaptation” were the functional expressions with the highest gene counts.

To further analyze the mechanism of action of the inter-root biotrophic bacteria, the differences in different metabolic functions of microorganisms at KEGG tertiary metabolic levels induced by the inter-root biotrophic bacteria were annotated (Figure 9B). Compared to CK, soil microorganisms in tobacco plants significantly increased fatty acid degradation and metabolic functions, glycine, serine, and threonine metabolic functions, lysine degradation functions, tryptophan metabolic functions, and tryptophan metabolic functions in the treatment of Bacillus paracolor in the T1 treatment. Arginine and proline metabolic functions, benzoic acid metabolic function, phenylalanine, tyrosine, and tryptophan biosynthetic functions were higher in the T1 treatment than in the control CK treatment. Amino acids are nutrients that ensure plant growth and development and maintain the life activities of soil microorganisms, and T1 promoted the biosynthesis function of amino acids in the soil microorganisms in the inter-root of the tobacco plant. Moreover, the synthesis of secondary metabolites of soil microorganisms was higher in the T1 treatment than in the CK treatment. T1 promoted the metabolic function of organic selenium compounds and the biosynthesis of cofactors compared with CK treatment.

Soil microorganisms are involved in material cycling and capacity flow in the soil, and to understand the metabolic functional properties of soil microorganisms involved in the dominant genera, a correlation analysis was conducted between the dominant bacteria in the soil and KEGG functions (Figure 9C). The correlation heat map revealed significant positive correlations between Gibberella, Chujaibacter, Trichoderma, and several functional categories, including membrane transport, drug resistance: antimicrobial, cellular community-prokaryotes, glycan biosynthesis, and metabolism, metabolism of other amino acids, metabolism of terpenoids and polyketides, and development and regeneration. Chujaibacter and Trichoderma also significantly and positively correlated with signal transduction and cell motility. Conversely, significant negative correlations were observed between Gibberella, Chujaibacter, and Trichoderma and cell growth and death, energy metabolism, translation, and nucleotide metabolism. Additionally, Chujaibacter and Trichoderma were significantly negatively correlated with carbohydrate metabolism, neurodegenerative diseases, the circulatory system, infectious diseases, and parasitic diseases.

## 4 Discussion

In this study, *Trichoderma harzianum*, a PGPG, was isolated from tobacco inter-root soils, and its mechanisms of action on continuously cropped soils were investigated. The results indicated that tobacco growth and development were significantly enhanced, and notable improvements in soil physicochemical properties were observed. Soil enzyme activities, microbial species, and functional diversity were further determined for the T1 treatment and CK. Sucrase, catalase, and urease levels were found to be significantly higher in the T1 compared to the CK. The T1 treatment increased beneficial microflora and promoted functions such as metabolism, amino acid synthesis, and secondary metabolite synthesis in soil microorganisms.

Agronomic traits are crucial prerequisites for expressing plant growth and quality, with plant growth and development directly indicated by these traits (Abdi and Williams, 2010). Agronomic traits and plant growth and development were found to be effectively enhanced by PGPR (Liu et al., 2023). For blueberries treated with the inter-root biotrophic bacteria *Pseudomonas* spp. and *Brucella* spp., significant increases were observed in the number of branches, leaves, chlorophyll content, and plant height compared to the control group (Lobato-Ureche et al., 2023). The growth of pepper seedlings was promoted by the inoculation of four indigenous strains of PGPR during their growth (Harahap et al., 2023). Significant increases in the number of grains, spikes, and root length were observed in rice following the application of PGPR (Abd El-Mageed et al., 2022). Studies have shown that adding *Bacillus mucilaginosus* to tobacco plants in the field positively affects plant height, maximum leaf length, maximum leaf width, and stem thickness. In the moderate addition group, the four indicators were 26.76, 15.24, 19.37, and 18.08% higher, respectively, compared to the control group (Zhao et al., 2024). In this study, treatment T1 significantly increased the height, maximum leaf length, maximum leaf width, and maximum leaf area of tobacco, consistent with the results of previous research.

Root development is directly linked to root vigor, which subsequently influences plant uptake and utilization of nitrogen and dry matter accumulation (Wang et al., 2019). The effects of inter-root biotrophic bacteria on crops are primarily manifested through their impact on the root system (Li et al., 2016). The morphology and structure of crop root systems influence their ability to absorb soil nutrients and water resources, subsequently affecting the growth of the above-ground parts (Lynch, 2013). A better root conformation increases plant access to water and nutrients in the growth substrate, improves plant water and fertilizer use efficiency, and enhances plant resistance (Mu et al., 2015; Bengough et al., 2011). Research shows that under salt stress, the application of PGPR significantly improves the root structure of oats, with root length increasing by 20.55 and 21.94% under 0 and 100 mM NaCl treatments, respectively (Zhang et al., 2023).

Additionally, PGPR from the *Bacillus* and *Enterobacter genera* has improved root architecture in wheat and maize, significantly enhancing root surface area, branching, length, and tips (Jochum et al., 2019). *Trichoderma harzianum*, which contains the cysteine-rich cell wall protein QID74, enhances root hair formation and elongation, increasing the absorption surface area and the efficiency of nutrient translocation to branches, thereby increasing plant biomass (Samolski et al., 2012). In this study, the root system was enhanced by T1 treatment. The total root length and the number of root tips of the root system were significantly increased by T1.

An increase in chlorophyll content was found to enhance the ability of plants to carry out photosynthesis significantly (Plus et al., 2005). Chlorophyll maintenance by *Bacillus* sp. was found to promote soybean growth (Mun et al., 2024). In barley, total chlorophyll was significantly increased by 126% by applying four different PGPRs (Slimani et al., 2023). The tomato was inoculated with *Bacillus subtilis* + *Bacillus amyloliquefaciens* and *Bacillus amyloliquefaciens*; increases in chlorophyll content were 30 and 27%, respectively. Chlorophyll b was 20 and 16%, respectively; total chlorophyll was 54 and 43%, respectively, and carotenoids were 52 and 42%, respectively (Gashash et al., 2022). In this study, treatment T1 increased the chlorophyll content in the leaves, enhancing photosynthesis and supporting tobacco growth. This is consistent with previous research. Biomass is considered an important parameter of vegetation status and is crucial for plant production, carbon cycling, and nutrient allocation studies. Previous studies using a PGPR isolate as an inoculum positively affected different saffron plant parameters. Enhancements were observed in the number of leaves and chlorophyll content. The yield of the underground part of cotyledons was significantly increased, approximately by 1.91 times, compared to the CK (Chamkhi et al., 2023). This study observed significant increases in root stems and leaves' fresh and dry weights following T1 treatment.

Soil enzyme activity plays a crucial role in the global cycling of key elements such as C, P, and N and serves as a vital indicator for assessing soil health and fertility levels (Daunoras et al., 2024). These activities, mainly produced through the physiological metabolism of various microbial groups, reflect the presence and activity of corresponding functional microorganisms. By studying these enzyme activities, researchers can assess microbial response mechanisms to environmental changes (Baldrian, 2009; Cusack et al., 2011).

For instance, the enzyme activity of soil SC is indicative of the soil's maturity and fertility levels, influencing the transformation of organic matter (OM) within the soil (Li et al., 2023). Soil catalase (CAT) activity, associated with soil microbial activity, aids plants in resisting oxidative damage (Khanna et al., 2019). Additionally, the degradation of nitrogen-containing compounds is effectively catalyzed by soil urease (UR), thereby maintaining soil health (Dutta and Neog, 2015). Previous studies have found that soil sucrase and urease activities, among others, were enhanced by the mixed inoculation of pastures with PGPR strains (Chai et al., 2023).

Significant improvements in soil sucrase, urease, and catalase activities were also observed following the application of Bacillus subtilis bacterial fertilizer to cigar soils (Shang et al., 2023). In this study, the effect of inter-root facultative bacteria on soil enzyme activities was analyzed, revealing that SC, CAT, and UR activities were significantly increased in the T1 treatments compared to the CK1 and CK2 controls, with the most pronounced differences observed in the T1 treatments. The T1 treatments not only enhanced the activities of SC, CAT, and UR in the soil, thereby boosting, but also corresponded with an increase in the OM and AN content in the inter-root soils. This suggests that the acceleration of microbial redox reactions and the metabolism of carbon and nitrogen in the soil are likely due to the T1 treatment.

Additionally, plant defenses are systemically activated by co-inoculation with *Trichoderma* and bacteria, involving both salicylic acid (SA)-related responses and jasmonic acid (JA)-related defense responses. For instance, a synergistic reduction in Erysiphe pisi conidial development on pea leaves was observed following root inoculation with *T. asperellum–P. fluorescens*, which was due to the systemic activation of JA-related defenses (Patel et al., 2016). This co-inoculation mechanism also enhances the activity of soil-related enzymes in plants, including phenylalanine ammonia-lyase (PAL), ascorbate peroxidase (APX), guaiacol peroxidase (GPX), and catalase (CAT) (Poveda and Eugui, 2022). Furthermore, urease (UR) plays a crucial role in characterizing the efficiency of urea utilization and its decomposition rate in the nitrogen cycle (Baligar et al., 1991). The addition of beneficial rhizosphere microorganisms has been shown to significantly increase the content of readily available nitrogen, thereby enhancing urea's conversion efficiency (Cookson and Lepiece, 1996).

The application of PGPR has been shown to alter the community structure of indigenous soil microorganisms to some extent. This alteration may be due to competition for limited soil nutrients and spatial distribution among indigenous microorganisms, or it may result from the promotion of characteristic secretions by plant roots, which recruit and induce beneficial microorganisms in the soil to transform soil nutrients (Li et al., 2021). Previous research has shown that applying *Trichoderma* significantly alters the number and diversity of native microbial populations, especially among bacteria. For example, in carrot crops, the application of *T. harzianum* significantly increased the populations of the *Bacillus* and *Pseudomonas* genera (Patkowska et al., 2020). Additionally, *T. harzianum* has shown high antagonism against pathogens, which is associated with the secretion of various proteins, including chitinase, mutanase, α-1,3-glucanase, α-1,2-mannosidase, carboxylic hydrolase ester, carbohydrate-binding module family 13, glucan 1,3-β-glucosidase, α-galactosidase, and neutral protease (de Lima et al., 2016).

After fumigation with Trichoderma, there was a significant increase in the abundance of beneficial microorganisms, particularly *Bacillus* and *Gemmatimonadaceae*. Both *Bacillus* and *Gemmatimonadaceae* are considered spectrum-beneficial bacteria and are recognized as spectrum-beneficial bacteria that can effectively enhance crop growth (Wu et al., 2022).

In this study, the application of T1 reduced the number of operational taxonomic units (OTUs) of bacteria and fungi in the soil. Regarding bacterial diversity, there was a reduction in species diversity and richness, while the relative abundance of dominant species increased with T1 treatment. Specifically, the relative abundance of dominant phyla, such as *Proteobacteria, Actinobacteria*, and *Candidatus Saccharibacteria*, was increased by T1 treatment. The phylum Proteobacteria is known for its complex physiological and metabolic capabilities, which play a crucial role in the carbon cycle (Liu, 2022). *Actinobacteria* participate in the metabolism of nitrogen and phosphorus in the soil, contributing to the decomposition of readily available phosphorus and nitrogen (Zeng et al., 2024). In addition, the relative abundance of *Ascomycota* and *Basidiomycota* in the fungal community was increased by T1 treatment.

As the main fungal decomposers in the soil, the fungal general within the *Ascomycota* phylum are predominantly saprophytes, which play a crucial role in the transformation of organic matter. Notably, the stammers within this group are particularly important in the decomposition of lignocellulose, a key structural component of plant cell walls (Yelle et al., 2008).

At the microbial genus level, the application of T1 treatment led to an increased relative abundance of dominant genera, including *Alcaligenaceae, Pandoraea*, and *Thiomonas*. The genus *Pandoraea* is recognized for its ability to mitigate drought stress and enhance growth traits in soybeans (Gonçalves et al., 2023). *Alcaligenaceae* has been shown to effectively promote growth and enhance salt resistance in canola (*Brassica napus* L.) (Abdel Latef et al., 2021). Additionally, the genes involved in the urea degradation process in *Thiomonas* strains have been reported to increase the rate of urea degradation in the soil, thereby enhancing soil fertility.

Urea degradation helps precipitate toxic metals such as iron, aluminum, and arsenic (Farasin et al., 2015). The T1 treatment also led to an increase in the relative abundance of *Hypocreaceae* and *Trichoderma* within the fungal community. Trichoderma, a member of Hypocreaceae, is known for its biocontrol and plant growth-promoting effects, including antimicrobial, antioxidant, and insecticidal activities, as well as growth enhancement (Lodi et al., 2023).

Soil microorganisms play an important role in cycling and transforming soil nutrients, root secretions, apoplastic materials, and other substances (Das et al., 2022). These functions, which help maintain a dynamic balance in the soil, are closely related to organic matter, mineral nutrients, and the life activities of microorganisms themselves (Fazeli-Nasab et al., 2022; Bhattacharyya et al., 2022). In this study, the application of T1 enhanced the metabolic functions of inter-root microorganisms in tobacco plants. This enhancement was observed in processes such as including amino acid biosynthesis and secondary metabolite synthesis. An increase was observed in metabolic activities such as membrane transport, drug resistance (antimicrobial), cellular community (prokaryotes), glycan biosynthesis and metabolism, other amino acid metabolism, terpenoids and polyketides, and development and regeneration induced by T1 treatment. Notably, the incidence of parasitic infectious diseases was reduced, indicating that the population of pathogenic bacteria in the soil was suppressed, leading to fewer diseases.

These changes in metabolic activities enhance the metabolism and energy flow of microorganisms in the soil (Khan et al., 2020; Yoo et al., 2021). In summary, T1 treatment can effectively improve soil properties and promote tobacco growth, providing a foundation for developing microbial fertilizers to alleviate continuous cropping barriers.

In this study, an efficient PGPR, *Trichoderma harzianum*, was isolated from the soil between tobacco roots, highlighting its potential in agriculture. This research provides a valuable reference for further exploration of fungal bioformulations and establishes a theoretical foundation for developing new fungal-centered microbial fertilizers, which can be integrated with existing agricultural management practices. However, this study has some limitations, including its confinements to a single type of soil, a lack of long-term research, and a focus on the application of a single strain. Therefore, future research should expand to include a broader range of crops and environments and conduct in-depth analyses of the economics and environmental impacts of long-term use to ensure the sustainable application of such bioformulation in modern agriculture.

## 5 Conclusion

This study successfully isolated *Trichoderma harzianum*, a PGPR strain with effective growth capabilities, from the soil between tobacco roots. The influence of this strain on the properties of soils subjected to continuous cropping was thoroughly examined. The results showed significant enhancements in the growth and development of tobacco plants, alongside notable improvements in the soil's physicochemical characteristics. A comparative analysis of soil enzyme activity, microbiome, and metagenome between the T1 treatment group and the CK revealed substantial increases in the activities of key enzymes such as sucrase, catalase, and urease in the T1 group. Furthermore, the T1 strain fostered an increase in beneficial microorganisms, which enhanced soil microbial functions related to amino acid metabolism, synthesis, and the production of secondary metabolites. These findings provide a strong foundation for the development of efficient microbial fertilizers with fungi at their core and contribute to the broader study of effective PGPR.

## Data Availability

The datasets presented in this study can be found in online repositories. The names of the repository/repositories and accession number(s) can be found here: the isolated strains genome can be found in the NCBI repository (https://www.ncbi.nlm.nih.gov/), accession number: PQ135054.
