# Supplementary material for: Isolation, identification, and mechanism analysis of plant growth-promoting rhizobacteria in tobacco
Source: Front Microbiol. 2024 Sep 20;15:1457624. doi: 10.3389/fmicb.2024.1457624 (PMC11449712; doi:10.3389/fmicb.2024.1457624)
Supplement: Supplementary file 1 [file Data_Sheet_1.docx]

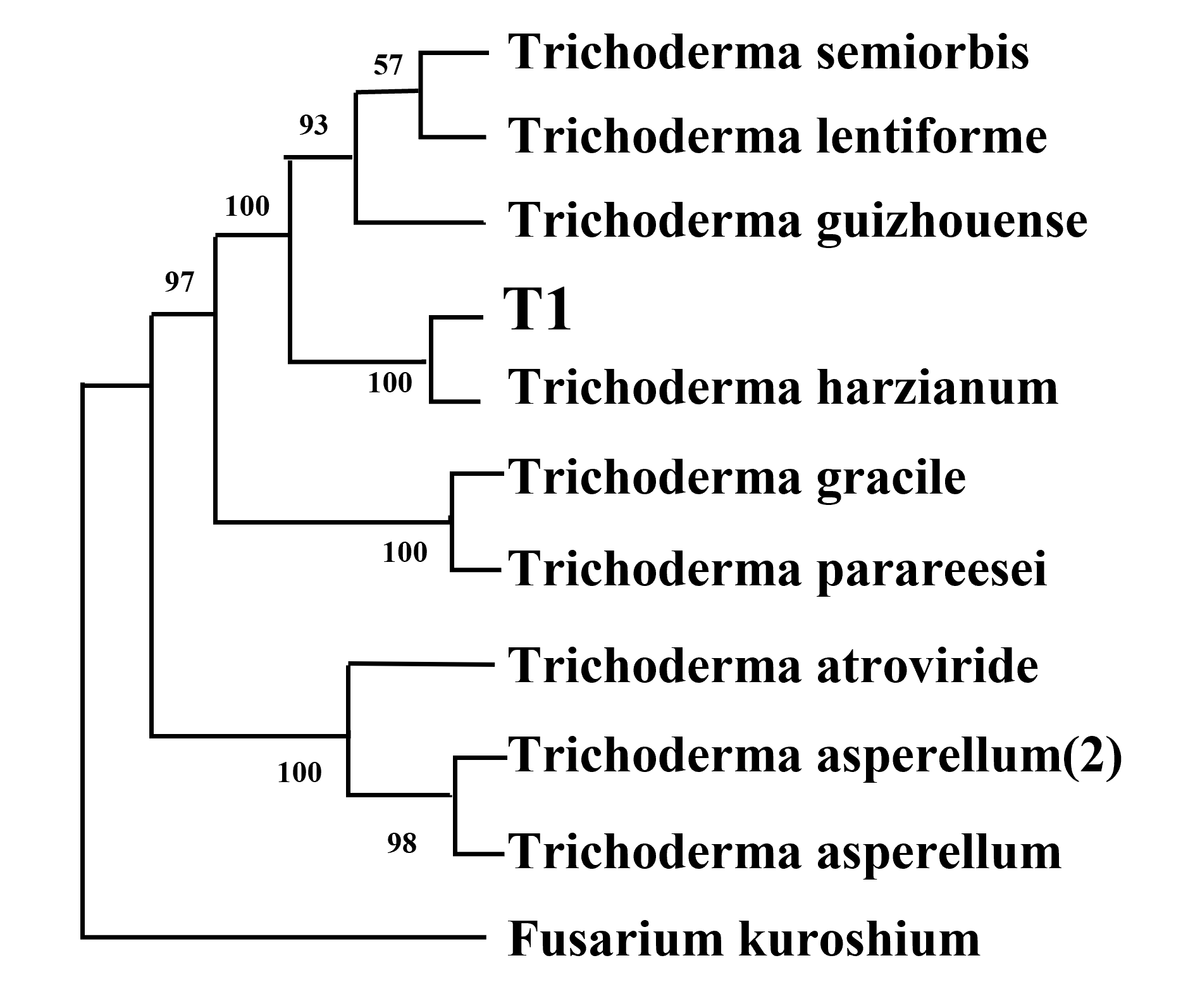


Figure S1 Phylogenetic tree based on 16S rDNA sequences.


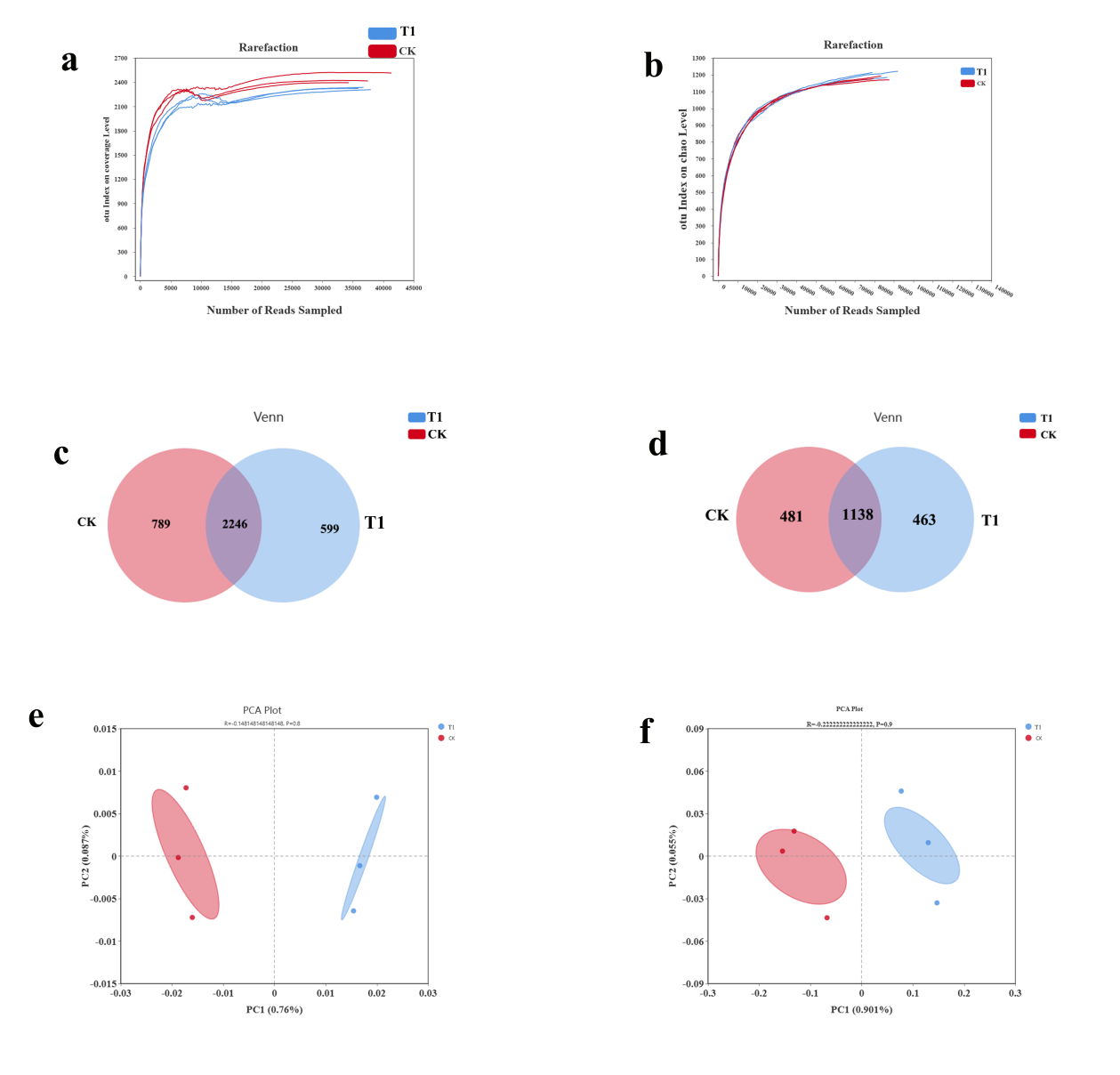


Figure S2 Dilution curves of different treatments based on OTU level coverage index (a) bacterial , (b)fungal. Venn diagrams of rhizosphere (c) bacteria, (d) fungi. PCA analysis of (e) bacterial, (f) fungal

Table S1 Characteristics of PGPR

| Number | Strains | Protein reduction | Dissolved inorganic phosphorus | Deoxidize organic phosphorus | Hemicellulose | Remove potassium | Produce IAA |
| --- | --- | --- | --- | --- | --- | --- | --- |
| T1 | *Trichoderma harzianum* | - | ++++ | +++ | + | ++ | ++ |

Notes: “+” represents the function was possessed.

“-” represents no function.
